# Synthesis and Luminescence Properties of Iridium(III) Azide- and Triazole-Bisterpyridine Complexes

**DOI:** 10.3390/molecules18088959

**Published:** 2013-07-26

**Authors:** Daniel C. Goldstein, Joshua R. Peterson, Yuen Yap Cheng, Raphael G. C. Clady, Timothy W. Schmidt, Pall Thordarson

**Affiliations:** 1 School of Chemistry, The University of New South Wales, NSW 2052, Australia; E-Mails: danielcgoldstein@gmail.com (D.C.G.); joshinoz2004@yahoo.com (J.R.P.); 2 School of Chemistry, The University of Sydney, NSW 2006, Australia; E-Mails: cheng_y@chem.usyd.edu.au (Y.Y.C.), clady@lp3.univ-mrs.fr (R.G.C.C.); timothy.schmidt@sydney.edu.au (T.W.S.)

**Keywords:** iridium(III), terpyridine, azide, click chemistry, lifetime measurements, ascorbate quenching

## Abstract

We describe here the synthesis of azide-functionalised iridium(III) bisterpyridines using the “chemistry on the complex” strategy. The resulting azide-complexes are then used in the copper(I)-catalysed azide-alkyne Huisgen 1,3-dipolar cycloaddition “click chemistry” reaction to from the corresponding triazole-functionalised iridium(III) bisterpyridines. The photophysical characteristics, including lifetimes, of these compounds were also investigated. Interestingly, oxygen appears to have very little effect on the lifetime of these complexes in aqueous solutions. Unexpectedly, sodium ascorbate acid appears to quench the luminescence of triazole-functionalised iridium(III) bisterpyridines, but this effect can be reversed by the addition of copper(II) sulfate, which is known to oxidize ascorbate under aerobic conditions. The results demonstrate that iridium(III) bisterpyridines can be functionalized for use in “click chemistry” facilitating the use of these photophysically interesting complexes in the modification of polymers or surfaces, to highlight just two possible applications.

## 1. Introduction

Iridium complexes have attracted much interest over the past several years due to their attractive luminescent properties [1,2]. Cyclometallated complexes of iridium that display high intensity luminescence are of special interest in the area of efficient organic light-emitting diodes (OLEDs) [3,4]. However, these complexes generally possess shorter lifetimes and higher oxygen sensitivity than iridium(III) bis-2,2':6',2"-terpyridine complexes which display outstanding luminescence properties with microsecond lifetimes and remarkable oxygen insensitivity for bioimaging [5].

These favourable properties result from the characteristic metal to ligand charge transfer (MLCT) state for Ir(III) bisterpyridine, which is at a high enough energy that it does not interfere with the emission properties. This is in stark contrast to other metal bisterpyridine complexes, for example ruthenium(II) terpyridines, which possess short excited-state lifetimes (0.25 ns) and negligible luminescence at room temperature [6] due to the presence of a MLCT state that is accessible *via* thermal pathways [7]. The high energy of the Ir(III) bisterpyridine excited state and long excited states lifetime have led to interest in using Ir(III) complexes in applications such as luminescent sensors and photovoltaics [5,8] This also makes the bisterpyridine complexes more suited to sensing applications where oxygen may be present, as well as for time-resolved and lifetime-based sensing methods [9,10,11].

Terpyridines are known to form complexes with a wide range of transition elements including first [Mn(II), Fe(II), Co(II), Ni(II), Cu(I), Zn(II)] [12], second [Mo, Ru(II), Rh(III), Pd(II), Cd(II)] and third [Re(II), Os(II), Ir(III), Pt(II), Au(III)] row d-block elements [13], group III [In(III)] as well as f-block elements [La, Pr, Nd, Sm, Eu(III), Gd, Tb, Dy, Ho, Er, Tm, Yb] [14]. Terpyridine complexes of the second and third row d-block elements, are much more kinetically stable than those of the first row d-block elements due to increased charge transfer between the ligand and metal [15]. It was only relatively recently that an efficient synthesis method for iridium terpyridine was developed [16]. We have adapted these methods for the synthesis of a series of novel complexes functionalised at the 4′-position and have reported previously on iridium(III) bisterpyridine complexes with aniline modifications at the 4′-position of the terpyridine [17]. These aniline complexes were of interest for sensing applications as the emission maximum shifts with solvent environment.

Carrying on from our previous results, we have explored the synthesis and photophysical properties of iridium(III) bisterpyridine complexes with benzyl azide modifications at the 4′-position of the terpyridine. The azide functionality is of particular interest in conjunction with “click” chemistry which has become a popular method for organic synthesis, particularly where benign conditions are required [18,19]. The most famous example, the copper(I)-catalysed azide-alkyne Huisgen 1,3-dipolar cycloaddition [19,20], is particularly attractive as it can be carried out in aqueous solutions with only catalytic copper(I) required and common functional groups (e.g., amines, carboxylic acids, thiols) do not interfere with the selective reaction. However, due the relatively harsh conditions required to synthesise iridium(III) bisterpyridines, azide- or alkyne-functionalised Ir(III) bisterpyridines have not been reported in the literature and subsequently they not found use in click chemistry.

Herein, we describe the synthesis of azide functionalized iridium(III) bisterpyridine complexes utilising the “chemistry on the complex approach” [21], the cycloaddition reaction with an alkyne compound of interest, and the photophysical properties of the resulting complexes. It is of interest to note that the triazol products of the cycloaddition reaction emit more strongly than the azide starting materials making them potentially useful in luminescent sensing applications.

## 2. Results and Discussion

### 2.1. Synthesis of Iridium(III) Bisterpyridine Azide Complexes ***5*** and ***6***

To synthesise the desired azide-functionalised iridium(III) bisterpyridine, the order of reactions, particularly the azide formation versus the crucial formation of the iridium(III) complex, needs to be carefully considered. Although iridium trichloride is the most obvious precursor for this complex formation, it is particularly inert. Ayala *et al.* reported the first iridium(III) bisterpyridine synthesis in 1990 based on melting the precursors together at elevated temperatures with arduous work-up [22]. Collin *et al.* then found that by refluxing iridium trichloride with the terpyridine precursors in ethylene glycol, yielded iridium(III) bisterpyridine in good yields with relative ease [16].

**Scheme 1:**
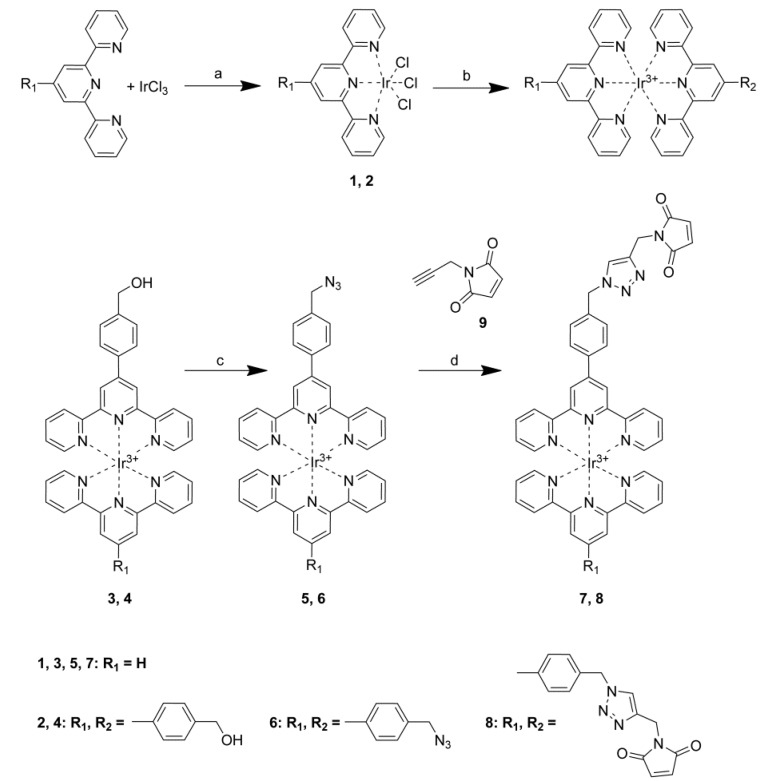
Step-wise synthesis of azide and triazolefuntionalisedIr(III) terpyridine complexes.

Previously reported attempts to prepare an azide-functionalised ruthenium complex by refluxing ruthenium(II) chloride and 4′-(4-azidomethyl)phenyl-2,2′:6′,2′′-terpyridine in ethanol have been unsuccessful, possibly due to the formation of side products as a result of azide coordination with ruthenium [23]. Since the conditions for iridium(III) complexation are even harsher, the direct introduction of an azide terpyridine was not attempted. The most common route for introducing an azide is by substitution of a suitable leaving group (bromine, tosyl) using sodium azide. However, attempts to form iridium(III) complexes using 4′-(4-bromomethylphenyl)-terpyridine were unsuccessful and lead to the reaction between the brominated terpyridine and the ethylene glycol solvent to result in an iridium(III) terpyridine complex functionalized instead with glycol groups. The successful synthesis of the ruthenium(II) 4′-(4-bromomethylphenyl)-terpyridine has been reported [24], but the yield for this reaction was only 5%, due to a similar side-reaction with ethanol at reflux. This illustrates the difficulty of introducing sensitive functional groups at the temperatures required for iridium(III) complexation. The iridium(III) terpyridine complexes described in this paper were therefore synthesised using the step-wise “chemistry on the complex” [21] strategy based on direct diphenyl phosphoryl azide activation of a 4-hydroxylmethylphenyl derivative as shown in Scheme 1 below.

The mono-complexes of Ir(III) **1**, **2** were synthesised by mixing iridium(III) chloride with the corresponding ligand and heating in degassed ethylene glycol at 160 °C for 15 min. After the reaction mixture had cooled, the resulting precipitate was filtered and washed with water, ethanol, and diethyl ether to give the mono-terpyridine complexes **1** and **2** in modest yields (39% and 47%, respectively). The resulting complexes are only soluble in dimethylsulfoxide and *N*,*N*-dimethylformamide. The structure and purity of the complexes were confirmed by HR-MS and ^1^H- and ^13^C-NMR. Further reaction of the mono-terpyridine complexes with the appropriate ligand in ethylene glycol at 160–180 °C for 20 min resulted in the bisterpyridine complexes **3** and **4** in reasonable yields (88% and 58%).

Thompson *et al.* have reported a direct conversion of activated alcohols to azides as a simple alternative to Mitsunobu conditions [25]. Following this procedure, an Ir(III) hydroxyl complex (**3** or **4**) was dissolved in a small amount of *N*,*N*-dimethylformamide to which was added 10 molar equivalents of diphenyl phosphoryl azide (Scheme 1). This mixture was cooled on ice before excess 1,8-diazabicyclo[5.4.0]undec-7-ene (DBU) was added dropwise to the stirring solution, and the reaction mixture was allowed to warm to room temperature and stir for several days. Addition of aqueous ammonium hexafluorophosphate resulted in orange to red precipitates that were filtered, washed with water, cold ethanol and diethyl ether. The reaction proceeds cleanly, with the only by-product being the water soluble DBU salt of diphenyl phosphate which remains in the aqueous solution or is washed off the precipated hexafluorophosphate salt of the complex. After recrystallisation from acetonitrile diethyl ether, pure azides **5** and **6** were obtained in yields up to 96%. The successful substitution of the alcohol with azide was then confirmed by the ^1^H-NMR shift of the methyl proton peak (0.2 ppm shift upfield), ^13^C-NMR, HR-MS and infrared spectra of the complexes showed a new peak corresponding to the characteristic azide stretch at 2100 cm^−1^.

*Reagents and Conditions*: (a) ethylene glycol, 160 °C, 15 min; (b) ethylene glycol, 160–180 °C, 20 min; (c) (C_6_H_5_)_2_P(O)N_3_ (diphenyl phosphorylazide), 1,8-diazabicyclo[5.4.0]undec-7-ene, *N,N*-dimethylformamide, room temperature, 3 days; (d) *N,N*-dimethylformamide, copper sulphate (aq), sodium ascorbate, 3 days.

### 2.2. Synthesis of 1-(Prop-2-ynyl)-1H-pyrrole-2,5-dione *(**9**)*

To assess the functionality of the azide group and its effect on the photophysical properties of the iridium complexes, an alkyne compound, 1-(prop-2-ynyl)-1*H*-pyrrole-2,5-dione [26] (**9**, Scheme 1), was synthesized for use in the “click” reaction. This compound was prepared for these and future studies due to its additional maleimide functionality, which can be used to selectively couple with thiol compounds through Michael addition [27]. The resulting alkyne-maleimide linker, is a key reagent that could be used to incorporate an alkyne group onto proteins containing cysteine residues, such as cytochrome *c* [28,29,30], or onto thiol-functionalized surfaces.

There are a number of reported routes for the synthesis of dione **9**, including the alkylation of a furan-protected maleimide with an alkyl halide under basic conditions [31], and the Mitsunobu reaction of the parent maleimide with an alcohol [32]. However, the classic method for dione synthesis is the condensation of maleic anhydride with a substituted amine, followed by ring closure in acidic conditions [33], and this was the route chosen for the synthesis of the previously reported dione **9** [26].

### 2.3. Click Chemistry Reaction

The azide complexes were subsequently reacted with alkyne-maleimide **9** using Cu(I) catalysed azide-alkyne cycloaddition. Azide complex **5** or **6** was combined with alkyne-maleimide **9** in *N*,*N*-dimethylformamide (1–2 mL) to which was added a small volume (0.2–1.0 mL) of a freshly prepared aqueous solution containing an excess of copper sulphate and sodium ascorbate (Scheme 1). After stirring for three days, excess aqueous ammonium hexafluorophosphate (30–40 mL) was added, precipitating yellow/orange solids which were recrystallised from acetonitrile/diethyl ether to give the products **7** and **8** in yields of 84% and 24%, respectively.

Formation of the products was confirmed by ^1^H- and ^13^C-NMR, HR-MS and infrared spectroscopy with the latter no longer shows the azide stretch at 2100 cm^−1^. The singlets in the deuterated acetonitrile ^1^H-NMR spectrum corresponding to the triazole (8.04 ppm), pyrolle (6.82 and 6.94 ppm) and methyl (adjacent to pyrolle) (5.73 and 5.83 ppm) integrate with the terpyridine protons in the correct ratios, but only if the delay time for the NMR acquisition is set to a suitably long enough value (*T_1_* = 20 s). This allows for differences in relaxation time between protons in the flexible triazole chain and on the terpyridine scaffold to be taken into account.

### 2.4. Steady-State Photophysical Characterization

**Figure 1:**
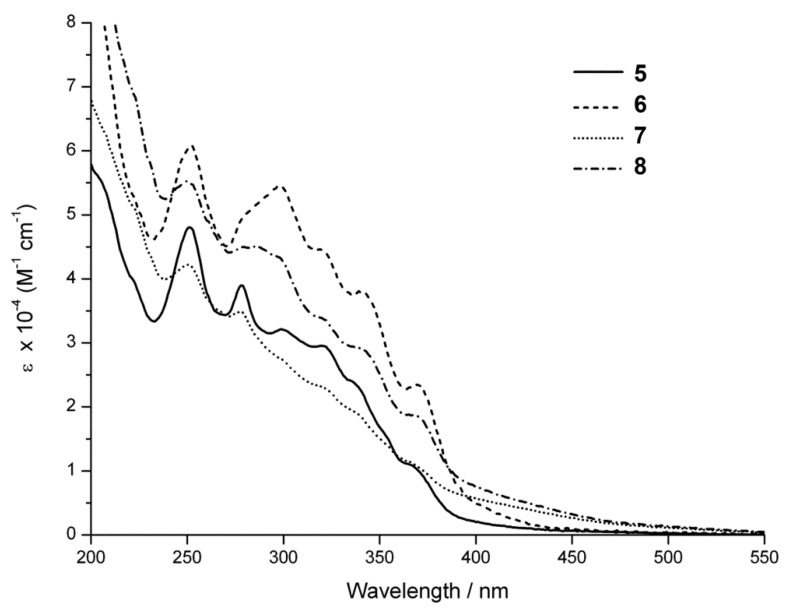
Room-temperature absorption spectra of acetonitrile solutions of **5**–**8**.

The absorption spectra of azido complexes **5** and **6** and triazoyl complexes **7** and **8** are shown in Figure 1. These complexes possess absorption properties typical of iridium bisterpyridine complexes, with a series of peaks appearing below 400 nm assigned to ligand centred transitions. The molar absorption coefficients are in the range of 10^4^ and 10^5^ M^−1^ cm^−1^ (Table 1), in agreement with previously reported values of related complexes.

All four complexes possess absorption tails extending into the visible region, and are more pronounced for triazoyls **7** and **8**. This extended absorption is evidence of ILCT processes similar to processes described previously for the related aniline complexes in which the functional group acts as an electron donor while the terpyridine acts as an acceptor [17]. This is also supported by the emission properties described below.

**Table 1:** Ground State Absorption Maxima and Intensities.

	λ_max_/nm (ε/M^−1^cm^−1^) ^a^
**5**	252 (47,900); 278 (39,000); 299 (32,100); 320 (29,500); 335 (24,200); 366 (10,900)
**6**	252 (61,000); 298 (54,600); 320 (44,600); 341 (37,800); 370 (23,400)
**7**	250 (42,200); 298 (34,800); 320 (23,200); 365 (11,400); 371 (26,300)
**8**	251 (55,100); 286 (45,000); 320 (33,800); 341 (29,000); 370 (18,600)

*^a^* CH_3_CN solvent.

**Figure 2:**
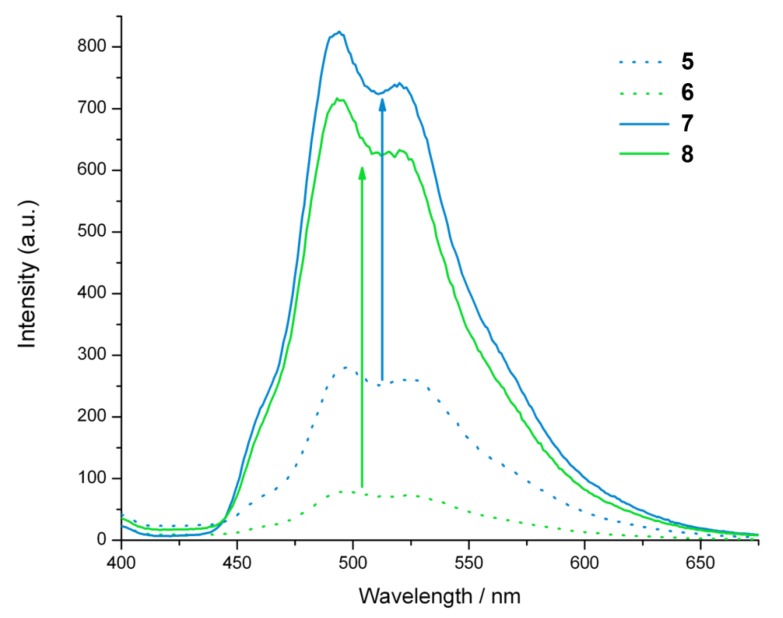
Room-temperature luminescence spectra of air-equilibrated iso-absorbing acetonitrile solutions of **5**–**8**. Excitation was at 350 nm. The arrows indicate the increase in emission intensity upon triazole formation.

Although there is little difference in the absorption spectra of these complexes, the emission obtained with the azide complexes **5** and **6** is much weaker than that of the triazole complexes **7** and **8**, and also weaker than the emission of related complexes possessing hydroxyl groups [17]. Furthermore, the bis-azide complex **6** produces the weakest emission, suggesting that the azide group provides the most efficient deactivation pathway (Figure 2). The substantial increase in emission intensity upon triazole formation suggests that the azide complexes **5** and **6** could be useful for the detection of alkyne-tagged proteins, as the increase in emission intensity would improve signal to noise against a background of “unbound” azide complexes. The increase in emission intensity could also be used to optimise the efficiency of copper catalysed cycloaddition reactions with organic alkynes.

### 2.5. The Effect of Ascorbate on Using Luminescence to Monitor the “Click” Reaction

**Figure 3:**
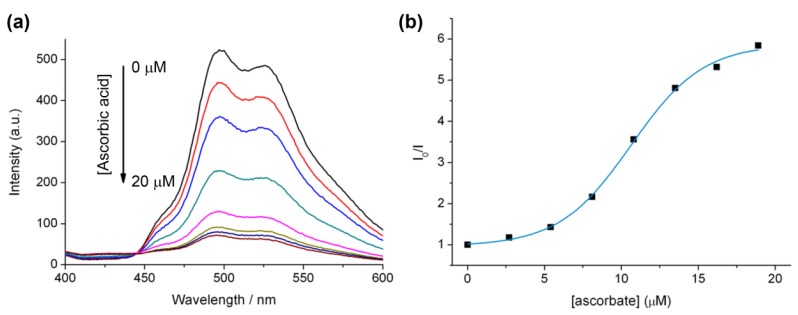
(**a**) Luminescent quenching of **7** upon the addition of ascorbate under aerated aqueous conditions at room temperature. Conditions, **7** (10 µM), HEPES buffer (20 mM) pH 7.0. (**b**) Decrease in λ_max_ (498 nm) for **7** (10 µM), HEPES buffer (20 mM) pH 7.0, as a function of added ascorbic acid (from 0 to 20 µM).

The optimisation of cycloaddition reactions is often carried out by monitoring reactions using azide dyes that improve in fluorescence or luminescence upon triazole formation [34], and the most frequently used catalysts for click reactions involve ascorbate reduction of copper(II) to copper(I) *in situ*. Monitoring the change in the luminescence of going from the Ir(III) bisterpyridine azide complex **5** to the corresponding triazole complex **7** under these conditions was complicated by the discovery that ascorbate is an efficient luminescent quencher of **7** under these conditions (Figure 3). Ascorbate is a biological antioxidant that quenches singlet oxygen [35], and is a well-known fluorescent quencher of other chromophores such as chlorophyll (triplet state) [36] and tryptophan [37,38].

The nonlinear behaviour observed in the Stern-Volmer plot (Figure 3B) suggests that quenching may be due to a combination of static and dynamic binding. This may be accounted for by electrostatic interactions between the negatively charged ascorbate and the tripositive Ir(III) complex.

**Figure 4:**
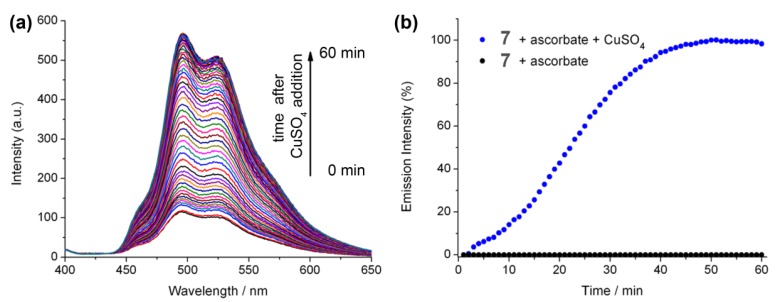
(**a**) Luminescence of **7** (10 μM), HEPES buffer (20 mM) pH 7.0 with ascorbate (40 μM) solution from *t* = 0 min to *t* = 60 min after the addition of copper(II)sulphate (40 µM). (**b**) Increase in λ_max_ (498 nm) for **7** (10 µM), HEPES buffer (20 mM) pH 7.0, as a function of time upon the addition of copper(II)sulphate (40 µM).

The effect of copper(II) sulphate addition to a mixture of Ir(III) complex **7** and ascorbate was also investigated, and resulted in a gradual increase in the emission intensity (Figure 4). This may be due to the consumption of oxygen in solution due to the oxidation of ascorbate in the presence of copper(II). In fact, such copper sulphate/ascorbate systems have previously been reported in relation to luminescent probes for oxygen intake in biological systems using the more oxygen-sensitive ruthenium bipyridine [39]. These two factors (ascorbate quenching and oxygen consumption) make monitoring the efficiency of the reaction under these conditions a difficult task and likely difficult to quantify.

### 2.6. Luminescent Lifetimes of the Iridium(III) Azide and Triazole Bisterpyridine Complexes

Generally, it has been found that the emission properties of iridium(III) bisterpyridine complexes are significantly worse in acetonitrile than in water. Reduced quantum yields (calculated from integration of the emission spectra on an energy scale) and shortened luminescent lifetime in acetonitrile are believed to be due to increased rates of non-radiative deactivation [5]. This makes the iridium(III) bisterpyridines more suited to aqueous conditions for application purposes.

The emission lifetimes of the azide and triazole complexes are substantially different from Ir(III) complexes described previously, especially with regards to lifetime in acetonitrile as compared to water (Table 2). Degassing aqueous solutions of the azide and triazole complexes has almost no effect on the emission lifetimes, and surprisingly long lifetimes are obtained in degassed acetonitrile. The reduction in quantum yield, the shorter lifetimes in degassed water and the longer lifetimes in degassed acetonitrile are indicative of emission from charge transfer bands similar to those described for the previously described aniline complexes [17]. In those cases, the lifetimes were typically 2 to 3 times (and up to 5 times) longer in degassed water than the lifetimes in degassed acetonitrile. However, for azide and triazole complexes **5**, **6** and **8**, the reverse is true with lifetimes in degassed acetonitrile being longer than the lifetimes in degassed water.

**Table 2:** ^a^Band maximum for the highest energy peak, uncorrected spectra. ^c^Quantum yields obtained from corrected spectra.

	CH_3_CN			H_2_O		
	λ^a^ (nm)	τ^b^ (μs)	10^2^ x Φ_e_^c^	λ^a^ (nm)	τ^b^ (μs)	10^2^ × Φ_e_^c^
**5**	498	1.5 (5.7)	0.7	497	3.8 (3.9)	0.9
**6**	498	1.3 (8.9)	0.5	497	4.0 (4.0)	0.3
**7**	493	1.3 (2.9)	1.4	494	3.6 (4.5)	2.5
**8**	495	2.3 (5.2)	2.1	493	2.3 (2.7)	2.2

^a^ Emission band maximum for the highest energy peak. ^b^ Emission lifetimes (λ_ex_ = 360 nm) obtained with air-equilibrated samples, values in parenthesis for degassed solvent, 293 K. ^c^ Quantum yields (λ_ex_ = 350 nm) obtained from corrected spectra using bis(4-tolyl-2,2′:6′:2′′-terpyridine)iridium(III)tris(hexafluorophosphate) at 293 K as 0.029 in acetonitrile as a reference [16]. Quantum yields in water are adjusted for refractive index [36].

## 3. Experimental

### 3.1. Chemicals

Chemicals were purchased from Sigma Aldrich (St. Louis, MO, USA) with the exceptions of ammonium acetate, sodium dihydrogen phosphate, sodium carbonate, sodium hydroxide, anhydrous sodium sulphate which were purchased from Ajax Finechem Pty. Ltd. (Sydney, Australia) 4-nitrobenzaldehyde (BDH, Poole Dorset, UK) and ammonium hexafluorophosphate (Acros Organics, Geel, Belgium). Neutral alumina oxide (alumina Oxide 90 neutral, 20–230 mesh) was purchased from Merck (city, country) and silica (Davisil 40–63 µM) was purchased from Grace (Deerfield, IL, U.S.A.). Dichloromethane and methanol were distilled before use. Dry solvents such as acetonitrile, dichloromethane, diethyl ether, and tetrahydrofuran were obtained from a Pure Solv dry solvent system (Innovative Technology, Inc. model #PS-MD-7, Amesbury, MA, USA). Dry *N*,*N*-dimethylformamide and dimethylsulfoxide were purchased from Sigma Aldrich and used directly from the bottle. Deuterated solvents for NMR were obtained from Cambridge Isotope Laboratories (Tewksbury, MA, USA).

### 3.2. Spectroscopy

^1^H-NMR spectra were recorded in the designated solvents using a Bruker Avance (200 MHz) or a Bruker Avance DPX (300 MHz) spectrophotometer. Multiplicities are assigned as singlet (s), doublet (d), triplet (t), quartet (q), multiplet (m) and denoted as broad (br) where appropriate. Chemical shifts are measured in parts per million (ppm), internally referenced relative to tetramethylsilane (SiMe_4_, ^1^H and ^13^C = 0 ppm) or residual solvent peaks (CD_3_CN: ^1^H = 1.94 and ^13^C = 1.32; DMSO-*d_3_*: ^1^H = 2.50, ^13^C = 39.52). UV-Vis spectra were recorded using a Varian Cary 5E UV-Vis-NIR spectrophotometer as solutions in spectroscopy-grade acetonitrile, distilled water, or buffer solution, as indicated in the text. Uncorrected data is reported as wavelength (λ) in nm and absorption coefficient (ε) in cm^−1^ M^−1^. Infrared spectra were recorded either on a Perkin-Elmer 580B or a Spotlight 400 FT-IR with an ATR module, and refer to solid sample by ATR or KBR disk, or as liquid sample in acetonitrile using a liquid IR cell as indicated in the text. MS (ESI) was carried out either on a Finnigan Mat SpectaSystem HPLC with a Finnigan LCQ-DECA electrospray mass spectrometry unit or a Waters Micromass ZQ 2000 ESCi Multi-mode Ionisation Source mass spectrometer. High resolution ESI mass spectra were recorded on a Thermo Scientific Linear Quadropole Ion Trap with Orbit Mass Analyser (LTQ ORBITRAP XL) mass spectrometer.

### 3.3. Steady-State and Lifetime Fluorescence

Room temperature fluorescence measurements were acquired using a Varian Cary Eclipse fluorescence spectrophotometer with either air-equilibrated or degassed samples as indicated. Degassing was performed by freeze-thawing (three cycles) in a degassing cuvette until pressure was less than 10^−2^ mmHg. Uncorrected luminescence band maxima are reported throughout the text. Luminescence quantum yields were obtained from corrected spectra using bis(4-tolyl-2,2′:6′:2′′-terpyridine)iridium(III)tris(hexafluorophosphate) at 293 K in acetonitrile as a reference (λ_ex_ = 350 nm, Φ_e__(__ref)_ = 0.029) [16]. Quantum yields in water were adjusted for refractive index (according to equation: Φ_e_ = Φ_e__(__ref)_ × (A_r_ × n^2^ × I)/(A × n_ref__)_^2^ × Ir) where A is absorbance (~0.07 AU) and n is refractive index of the solvent) [40]. The quantum yields are based on the area under the emission curve plotted on an energy rather than wavelength scale. Luminescence lifetimes were obtained using a femtosecond Clark MXR CPA 2210 laser source equipped with a Topaz (-c) light converter to obtain excitation light at 380 nm. The emission was recorded by a Spectrapro 2300i monochromator (Princeton Instruments, Trenton, NJ, USA) with attached iCCD camera. The uncertainty of the evaluated lifetimes is 20%.

### 3.4. Syntheses

#### 3.4.1. General Method A: Preparation of Ir(terpy-R)Cl_3_

Iridium(III) trichloride hydrate salt (73–205 mg, 0.244–0.948 mmol) and the desired terpyridine (64–224 mg, 0.274–0.691 mmol, 1.1 mol. eq.) were crushed together with a glass rod. Ethylene glycol (5–15 mL) was added and the mixture degassed with three vacuum/nitrogen cycles before being heated to 160 °C in the dark and under nitrogen atmosphere for 15 min. The reaction mixture was allowed to cool to room temperature and the resulting precipitate was collected by filtration and washed with water, ethanol, and diethyl ether.

#### 3.4.2. General Method B: Preparation of [Ir(terpy-R^1^)(terpy-R^2^)](PF_6_)_3_

Iridium(III) trichloride hydrate (20–147 mg, 0.041–0.236 mmol) salt and the corresponding terpyridine (22–150 mg, 0.041–0.669 mmol, 1.1 mol. eq.) were crushed together with a glass rod. Ethylene glycol (5–15 mL) was added and the mixture degassed with three vacuum/nitrogen cycles before being heated to 160–180 °C in the dark and under nitrogen for 20 min. The reaction mixture was allowed to cool to room temperature before an aqueous solution of excess ammonium hexafluorophosphate (40–100 mL) was added. The resulting precipitate was collected by filtration and washed with water, ethanol, and diethyl ether, followed by recrystallisation from acetonitrile/diethyl ether. If necessary, this was then purified by column chromatography followed by precipitation using aqueous ammonium hexafluorophosphate, filtered, and recrystallised from acetonitrile/diethyl ether to give the final product.

#### 3.4.3. General Method C: Direct Conversion of Alcohols to Azides Using Diphenyl Phosphorazidate (S_N_2)

A solution of metal terpyridine complex [possessing one or two 4′-(4-hydroxymethylphenyl)-2,2′:6′2′′-terpyridine ligands: M(terpy-Φ-CH_2_OH)_n_(terpy)_(2-n)_ (where n = 1,2, M = Ir, Ru)] and diphenyl phosphoryl azide (DPPA, 10 mol. eq.) in dry dimethylformamide (1–2 mL) was cooled to 0 °C for 10 min before 1,8-diazabicyclo[5.4.0]undec-7-ene (DBU, 12 mol. eq.) was added dropwise and the reaction mixture allowed to warm to room temperature. The reaction was left stirring in the dark and under nitrogen for 3 days before an aqueous solution of ammonium hexafluorophosphate (30–40 mL) was added to precipitate a solid that was collected by filtration and recrystallised from acetone/diethyl ether to yield the product.

#### 3.4.4. General Method D: Copper Catalysed Huisgen 1,3-Dipolar Cycloaddition

To a solution of metal terpyridine complex [possessing either one or two 4′-(4-azidomethylphenyl)-2,2′:6′2′′-terpyridine ligands: M(terpy-Φ-CH_2_N_3_)_n_(terpy)_(2-n)_ (where n = 1,2, M = Ir, Ru)] and 1-(2-propynyl)-1*H*-pyrrole-2,5-dione **9** in *N,N*-dimethylformamide (1–2 mL), an aqueous solution (200–1,000 μL) of copper sulphate (3–10 mol. eq.) and sodium ascorbate (3–10 mol. eq.) was added and the solution stirred for 3 days in the dark and under nitrogen. Aqueous ammonium hexafluorophosphate (30–40 mL) was then added and the resulting precipitate collected by filtration and recrystallised from acetonitrile:diethyl ether to give the final product.

*Synthesis of 4′-(4-Hydroxymethylphenyl)-2,2′:6′2′′-terpyridine* starting material [41]. To a mixture of crushed sodium hydroxide (1.00 g, 25.0 mmol) and poly(ethylene glycol) (PEG-400) (15 mL) at 0 °C was added 2-acetylpyridine (3.78 g, 31.0 mmol). After 5 min, 4-(hydroxymethyl)benzaldehyde (**2**, 1.80 g, 13.0 mmol) was added and stirred at 0 °C for 2 h. Aqueous ammonia (15 mL, 25%) was then added and was heated to 100 °C for an additional 2 h. The resulting precipitate was collected by filtration and washed with water and cold ethanol. Recrystallisation from ethanol gave the terpyridine 4′-(4-Hydroxymethylphenyl)-2,2′:6′2′′-terpyridine as an off-white solid (1.50 g, 34%): mp 203–204 °C; ^1^H-NMR (200 MHz, CDCl_3_): δ 8.74–8.66 (m, 6H, H_3′_, H_6_, H_3_), 7.93–7.84 (m, 4H, H_4_, H_5_), 7.51 (d, 2H, *J* = 8.5 Hz, H_b_), 7.35 (ddd, 2H, *J* = 7.2, 5.5, 1.7 Hz, H_a_), 4.80 (d, 2H, *J* = 5.4 Hz, -C*H_2_*OH), 1.84 (br s, 1H, -OH). MS (ESI): *m/z* 340.18 ([M+H]^+^, C_22_H_18_N_3_O^+^ requires 340.15). This data is in agreement with reported literature [41].

*Synthesis of trichloro(2,2′:6′:2′′-terpyridine)iridium(III)* (**1**) [16]. Using general method A, iridium(III) trichloride hydrate (106 mg, 0.274 mmol) salt and 2,2′:6′2′′-terpyridine (64 mg, 0.274 mmol) yielded complex **1** as a red solid (58 mg, 39%): mp >300 °C; ^1^H-NMR (200 MHz, DMSO-*d_6_*): δ 9.21 (d, 2H, *J* = 4.5 Hz, H_6_), 8.77–8.69 (m, 4H, H_3′_, H_3_), 8.31–8.18 (m, 3H, H_4′_, H_4_), 7.99–7.93 (dd, 2H, *J* = 6.6 Hz, H_5_). MS (ESI): *m/z* ([M−Cl]^+^, C_15_H_11_IrN_3_Cl_2_^+^ requires 496.0). FT-IR (ATR): *υ*/cm^−1^ 3443br, 3063w, 2923w, 2853w, 1602s, 1546w, 1473m, 1434m, 1393m, 1237m, 1159w, 1104m, 1049m, 819m, 782s. This data is in agreement with reported literature [16].

*Synthesis of trichloro(4′-(4-hydroxymethylphenyl)-2,2′:6′2′′-terpyridine)iridium(III)* (**2**). Using general method A, iridium(III) trichloride hydrate (100 mg, 0.335 mmol) salt and 4′-(4-hydroxymethylphenyl)-2,2′:6′2′′-terpyridine (115 mg, 0.339 mmol) yielded complex **2** as a red solid (100 mg, 47%): mp > 300 °C; ^1^H-NMR (300 MHz, DMSO-*d_6_*): δ 9.22 (dd, 2H, *J* = 5.6, 1.0 Hz, H_6_), 9.09 (s, 2H, H_3′_), 8.90 (d, 2H, *J* = 9.0 Hz, H_3_), 8.29 (ddd, 2H, *J* = 7.9, 7.9, 1.5 Hz, H_4_), 8.19 (d, 2H, *J* = 8.34 Hz, H_a_), 7.97 (ddd, 2H, *J* = 6.9, 5.7, 1.3 Hz, H_5_), 7.61 (d, 2H, *J* = 8.4 Hz, H_b_), 5.38 (t, 1H, *J* = 5.6 Hz, -O*H*), 4.67 (s, 2H, -C*H_2_*OH). ^13^C (75 MHz, DMSO-*d_6_*): δ 159.2, 157.3, 153.0, 150.9, 145.9, 140.3, 133.3, 128.4, 128.0, 127.0, 125.4, 120.7, 62.8, 62.4. HRMS (ESI): *m/z* 659.9913 ([M+Na]^+^ C_22_H_17_N_3_ONaCl_3_Ir^+^ requires 659.9964). MS (ESI): m/z 660.2 ([M+Na]^+^ C_22_H_17_N_3_ONaCl_3_Ir^+^ requires 660.0). FT-IR (ATR): υ/cm^−1^ 3509w, 3067w, 2857w, 1697w, 1605s, 1545m, 1471m, 1427m, 1405s, 1300w, 1236w, 1155w, 1051m, 1011w, 977w, 885w, 811w, 786s.

*Synthesis of* *(4′-(4-hydroxymethylphenyl)-2,2′:6′2′′-terpyridine)(2,2′:6′:2′′-terpyridine)iridium(III)tris (hexafluorophosphate)* (**3**). Using general method B, trichloro(2,2′:6′:2′′-terpyridine)iridium(III) **1** (100 mg, 0.183 mmol) and 4′-(4-hydroxymethylphenyl)-2,2′:6′2′′-terpyridine (67 mg, 0.198 mmol), after purification by column chromatography (silica, acetonitrile/water/aqueous potassium nitrate (saturated), 70:29:1, v/v/v), yielded complex **3** (195 mg, 88%): mp >300 °C; ^1^H-NMR (300 MHz, CD_3_CN): δ 9.07 (s, 2H, H_3′_ of **3**), 8.85 (d, 2H, *J* = 8.8 Hz, H_3′_ of **1**), 8.80–8.70 (m, 3H, H_3_ of **3**, H_4′_ of **1**), 8.59 (dd, 4H, *J* = 7.5, 0.7 Hz, H_3_ of **1**), 8.26–8.18 (m, 6H, H_4_, H_b_ of **3**, H_4_ of **1**), 7.77 (d, 2H, *J* = 7.7 Hz, H_a_ of **3**), 7.69 (dd, 2H, *J* = 5.7, 0.8 Hz, H_6_ of **3**), 7.58 (dd, 2H, *J* = 4.3, 0.5 Hz, H_6_ of **1**), 7.52–7.45 (m, 4H, H_5_ of both terpys), 4.82 (s, 2H, -C*H_2_*-). ^13^C (75 MHz, CD_3_CN): δ 159.0, 157.0, 155.7, 155.5, 154.4, 154.3, 144.7, 143.9, 134.8, 130.8, 129.4, 128.7, 128.5, 127.6, 125.1, 64.0. HRMS (ESI): *m/z* 1055.1226 ([M−PF_6_]^+^, C_37_H_28_F_12_IrN_6O_P_2_ requires 1055.1237). MS (ESI): *m/z* 1055.21 ([M−PF_6_]^+^, C_37_H_28_F_12_IrN_6_OP_2_ requires 1055.12). FT-IR (ATR): *υ*/cm^−1^ 3418br, 3063w, 2939w, 2865w, 1606s, 1548m, 1474m, 1403m, 1241m, 1105m, 1052m, 836s (PF_6_).

*Synthesis of bis(4′-(4-hydroxymethylphenyl)-2,2′:6′:2′′-terpyridine)iridium(III) tris(hexafluorophosphate)* (**4**). Using general method B, trichloro(4′-(4-hydroxymethylphenyl)-2,2′:6′2′′-terpyridine)iridium(III) **2** (100 mg, 0.157 mmol) and 4′-(4-hydroxymethylphenyl)-2,2′:6′2′′-terpyridine (58 mg, 0.171 mmol) yielded complex **4** as an orange-yellow solid (120 mg, 58%): mp >300 °C; ^1^H-NMR (300 MHz, CD_3_CN): δ 9.09 (s, 4H, H_3′_), 8.73 (d, 4H, *J* = 7.9 Hz, H_3_), 8.27–8.19 (m, 8H, H4, H_b_), 7.77 (d, 4H, *J* = 8.31 Hz, H_a_), 7.70 (d, 4H, *J* = 4.5 Hz, H_6_), 7.50 (dd, 4H, H_5_, coupling constants too close to allow exact values), 4.83 (s, 4H, -C*H_2_*OH), 3.50 (br s, 2H, O*H*). ^13^C-NMR (75 MHz, CD_3_CN): δ 159.0, 156.9, 155.4, 154.2, 147.6, 143.7, 134.7, 130.6, 129.3, 128.6, 128.2, 125.0, 63.9. HRMS (ESI): *m/z* 1161.1665 ([M−PF_6_]^+^, C_44_H_34_F_12_IrN_6_O_2_P_2_^+^ requires 1161.1655). MS (ESI): *m/z* 1161.1 ([M−PF_6_]^+^ requires 1161.2). IR (KBr) *υ*/cm^−1^: 3675m, 3110m, 2950w, 2875w, 2360m, 2339m, 1700s, 1653m, 1558m, 1479m, 1250m, 838s (PF_6_).

*Synthesis of* *(4′-(4-azidomethylphenyl)-2,2′:6′,2′′-terpyridine) (2,2′:6′,2′′-terpyridine)iridium(III)tris(hexafluorophosphate)* (**5**). Using general method C, (4′-(4-hydroxymethylphenyl)-2,2′:6′:2′′-terpyridine) (2,2′:6′,2′′-terpyridine)iridium(III)tris(hexafluorophosphate) **3** (190 mg, 0.156 mmol), diphenylphosphorylazide (DPPA) (444 mg, 0.366 mmol), and 1,8-diazabicyclo[5.4.0]undec-7-ene (DBU) (234 mg, 1.54 mmol) yielded complex **5** as a dark red solid (184 mg, 96%). ^1^H-NMR (300 MHz, CD_3_CN): δ 9.08 (s, 2H, H_3′_ of terpy*-*N_3_), 8.86 (d, 2H, *J* = 8.0 Hz, H_3′_ of **1**), 8.80–8.69 (m, 3H, H_3_ of terpy*-*N_3_, H_4′_ of **1**), 8.59 (d, 4H, *J* = 8.0 Hz, H_3_ of **1**), 8.25–8.18 (m, 6H, H_4_ and H_b_ of terpy*-*N_3_, H_4_ of **1**), 7.78 (d, 2H, *J* = 8.1 Hz, H_a_ of terpy*-*N_3_), 7.68 (d, 2H, *J* = 5.3 Hz, H_6_ of terpy*-*N_3_), 7.59 (d, 2H, *J* = 5.2 Hz, H_6_ of **1**), 7.51–7.49 (m, 4H, H_5_ of **1** and H_5_ of terpy*-*N_3_), 4.65 (s, 2H, -C*H_2_*-). ^13^C (75 MHz, CD_3_CN): δ 158.9, 155.7, 155.6, 154.5, 154.3, 144.7, 143.8, 134.8, 130.8, 130.6, 129.9, 128.4, 127.6, 125.3, 54.6. HRMS (ESI): *m/z* 1080.1305 ([M−PF_6_]^+^, C_37_H_27_F_12_IrN_9_P_2_^+^ requires 1080.1302). FT-IR (ATR): *υ*/cm^−1^ 3647w, 3092w, 2102m (N_3_), 1609m, 1480m, 1456m, 1405w, 1250m, 1034m, 825s (PF_6_).

*Synthesis of bis(4′-(4-azidomethylphenyl)-2,2′:6′,2′′-terpyridine)iridium(III)tris(hexafluorophosphate)* (**6**). Using general method C, bis(4′-(4-hydroxymethylphenyl)-2,2′:6′:2′′-terpyridine)iridium(III)tris(hexafluorophosphate) **4** (75 mg, 0.055 mmol), diphenylphosphorylazide (DPPA) (125 µL, 0.580 mmol), and 1,8-diazabicyclo[5.4.0]undec-7-ene (DBU) (100 µL, 0.670 mmol) yielded complex **6** as red crystals (55 mg, 70%). ^1^H-NMR (300 MHz, acetone-*d_6_*): δ 9.10 (s, 4H, H_3′_), 8.72 (d, 4H, *J* = 7.8 Hz, H_3_), 8.26–8.21 (m, 8H, H_4_ and H_b_), 7.80 (d, 4H, *J* = 8.4 Hz, H_a_), 7.70 (d, 4H, *J* = 5.2 Hz, H_6_), 7.61 (dd, 4H, H_5_, coupling constants too close to allow determination of their exact values), 4.65 (s, 4H, -C*H_2_*N_3_). ^13^C-NMR (75 MHz, CD_3_CN): δ 159.0, 156.5, 155.6, 154.4, 143.8, 141.1, 136.3, 130.6, 129.9, 128.4, 125.3, 54.6. HRMS (ESI): *m/z* 1211.1806 ([M−PF_6_]^+^, C_44_H_32_F_12_IrN_12_P_2_^+^ requires 1211.1786). MS (ESI): *m/z* 1211.28 ([M−PF_6_]^+^, C_44_H_32_F_12_IrN_12_P_2_^+^ requires 1211.18). IR (KBr) *υ*/cm^−1^: 3629w, 3110w, 2361w, 2336w, 2097m (s, N_3_), 1684m, 1653m, 1558m, 1506m, 1479m, 1437m, 1254m, 1033m, 1016m, 838s (PF_6_).

*Synthesis of (1-((1-(4-(2,2':6',2"-terpyridine)benzyl)-1H-1,2,3-triazol-4-yl)methyl)-1H-pyrrole-2,5-dione)(2,2':6',2''-terpyridine)iridium(III)tris(hexafluorophosphate)* (**7**). Using general method D, (2,2':6',2''-terpyridine)(4'-(4-azidomethylphenyl)-2,2':6',2''-terpyridine)iridium(III)tris(hexafluorophosphate) **5** (30 mg, 0.0245 mmol), 1-(2-propynyl)-1H-pyrrole-2,5-dione **9** (3.5 mg, 0.026 mmol), copper sulphate (30 mg, 0.044 mmol) and ascorbic acid (22 mg, 0.057 mmol) gave complex **7** as a yellow powder (28 mg, 84%): mp > 300 °C; ^1^H-NMR (300 MHz, CD_3_CN): δ 9.05 (s, 2H, H_3′_ of terpy-triazole), 8.86 (dd, 2H, *J* = 8.2, 1.9 Hz, H_3′_ of terpy), 8.77 (dd, 1H, *J* = 8.1, 2.6 Hz, H_4′_ of terpy), 8.68 (dd, 2H, *J* = 7.8, 0.6 Hz, H_6_ of terpy-triazole), 8.58 (dd, 4H, *J* = 8.1, 0.7 Hz, H_6_ of terpy), 8.25–8.17 (m, 6H, H_5_, H_a_ of **3**-Az, H_4_ of terpy), 7.69–7.66 (m, 4H, H_b_, H_3_ of terpy-triazole), 7.58 (dd, 2H, *J* = 5.7, 1.0 Hz, H_3_ of terpy), 7.50–7.45 (m, 4H, H_5_ of terpy and H_4_ of terpy-triazole), 6.82 (s, 2H, 2C*H* pyrrole), 5.73 (s, 2H, -C*H*_2_-), 4.75 (s, 2H, -C*H*_2_-). ^13^C (75 MHz, CD_3_CN): δ 158.90, 155.70, 155.56, 154.46, 154.28, 144.69, 143.84, 134.79, 130.78, 130.59, 129.88, 128.38, 127.59, 125.32, 54.58, 33.65. HRMS (ESI): *m/z* 1215.1636 ([M−PF_6_]^+^, C_44_H_32_F_12_IrN_10_O_2_P_2_^+^ requires 1215.1623). FT-IR (ATR): *υ*/cm^−1^ 3542br, 3091w, 2262s, 1715w, 1632w, 1483w, 1437w, 1193w, 1036m, 846s (PF_6_).

*Synthesis of bis(1-((1-(4-(2,2':6',2"-terpyridine)benzyl)-1H-1,2,3-triazol-4-yl)methyl)-1H-pyrrole-2,5-dione)iridium(III)tris(hexafluorophosphate)* (**8**). Using general method D, bis(4′-(4-azidomethylphenyl)-2,2*'*:6*'*,2*''*-terpyridine)iridium(III)tris(hexafluorophosphate) **6** (20 mg, 0.015 mmol), 1-(2-propynyl)-1H-pyrrole-2,5-dione **9** (10 mg, 0.074 mmol), copper sulphate (4.3 mg, 0.17 mmol) and ascorbic acid (2.5 mg, 0.142 mmol) gave complex **8** as a yellow solid (6 mg, 24%). m.p. >300 °C. ^1^H-NMR (300 MHz, acetone-*d_6_*): δ 9.56 (s, 4H, H_3′_), 9.16 (d, 4H, *J* = 7.9, H_6_), 8.42 (dd, 4H, H_5_, coupling constants too close to allow determination of their exact values), 8.29 (d, 4H, *J* = 8.2 Hz, H_a_), 8.22 (d, 4H, *J* = 4.5 Hz, H_3_), 8.04 (s, 2H, -NC*H*C- of triazole), 7.72 (d, 4H, *J* = 8.6 Hz, H_b_), 7.64 (dd, 4H, H_4_, coupling constants too close to allow determination of their exact values), 6.94 (s, 4H, 2C*H* pyrrole), 5.83 (s, 4H, C_6_H_4_-C*H_2_*-N-), 4.79 (s, 4H, C-C*H_2_*-N-). ^13^C-NMR (75 MHz, CD_3_CN): 207.81, 171.58, 158.99, 156.37, 155.60, 154.33, 143.76, 140.72, 136.35, 135.54, 130.77, 130.22, 128.33, 125.39, 124.35, 53.96, 33.65. HRMS (ESI): *m/z* 1481.2440 ([M−PF_6_]^+^, C_58_H_42_F_12_IrN_14_O_4_P_2_^+^ requires 1481.2426). MS (ESI): *m/z* 1480.8 ([M−PF_6_]^+^, C_58_H_42_F_12_IrN_14_O_4_P_2_^+^ requires 1481.2). FT-IR (ATR): *υ*/cm^−1^ 3636w, 3457br, 2263m, 1710m, 1611m, 1436m, 1409m, 1333m, 1145m, 1053m, 1034m, 832s (PF_6_).

*Synthesis of 1-(2-propynyl)-1H-pyrrole-2,5-dione* (**9**) [26,42]. Following a two-step process, propargylamine (0.56 g, 10.2 mmol) was added drop-wise to a solution of maleic anhydride (1.00 g, 10.2 mmol) in glacial acetic acid (20 mL), and the mixture was stirred overnight at room temperature in the dark. The solution was concentrated *in vacuo* to give a brown residue that was recrystallised from isopropyl alcohol/water (80:20, v/v) to give 4-oxo-4-(2-propyn-1-ylamino)-2-butenoic acid as long white crystals (354 mg, 23%). ^1^H-NMR (200 MHz, DMSO-*d_6_*): δ 9.17 (br s, 1H, -N*H*-), 6.28 (d, 2H, *J* = 6.0 Hz, C*H*=C*H*), 3.98 (dd, 2H, *J* = 4.4, 3.7 Hz, -C*H_2_*-), 3.19 (s, 1H, ≡C*H*). Then a mixture of 4-oxo-4-(2-propyn-1-ylamino)-2-butenoic acid (350 mg, 2.30 mmol) and sodium acetate (0.103 g, 1.25 mmol) in acetic anhydride (5 mL) was heated to 65 °C for 2 h in the dark. After cooling, the suspension was poured into ice cold water (30 mL) and extracted with diethyl ether (3 × 30 mL). The organic phase was dried over anhydrous sodium sulphate, filtered, and concentrated *in vacuo* to give a crude solid which was purified by column chromatography (silica, hexane/ethyl acetate, 50:50 v/v) to give 1-(2-propynyl)-1H-pyrrole-2,5-dione **9** as a pale yellow solid (70 mg, 22%). ^1^H-NMR (200 MHz, CDCl_3_): δ 6.77 (s, 2H, -C*H*=C*H*-), 4.30 (d, 2H, *J* = 2.5 Hz, -C*H_2_*-), 2.21 (t, 1H, *J* = 2.5 Hz, ≡C*H*). MS (EI): *m/z* 136.04 ([M+H]^+^, C_7_H_6_NO_2_^+^ requires 136.16). This data is in agreement with reported literature [26,42].

## 4. Conclusions

Azide functionalized iridium(III)bisterpyridine complexes were synthesized *via* a “chemistry on the complex” approach, and click chemistry was used form triazoyl maleimide complexes which could be used for the selective modification of thiols. The photophysical study of the azide and triazoyl complexes has illustrated the effects of introducing different substituents at the 4′-position of the terpyridine, which results in unusual emission properties due to an intraligand charge transfer process. In particular, the long excited state lifetime in degassed acetonitrile as compared to water is not typically seen for iridium terpyridine complexes. Additionally, the significant increase in emission upon triazoyl formation makes these complexes of interest for applications in luminescent sensing, e.g. for the functionalization of polymers or surfaces.
